# Hydro- and Icephobic Properties and Durability of Epoxy Gelcoat Modified with Double-Functionalized Polysiloxanes

**DOI:** 10.3390/ma16020875

**Published:** 2023-01-16

**Authors:** Katarzyna Ziętkowska, Rafał Kozera, Bartłomiej Przybyszewski, Anna Boczkowska, Bogna Sztorch, Daria Pakuła, Bogdan Marciniec, Robert Edward Przekop

**Affiliations:** 1 Faculty of Materials Science and Engineering, Warsaw University of Technology, ul. Woloska 141, 02-507 Warszawa, Poland; 2 Technology Partners Foundation, ul. Pawinskiego 5A, 02-106 Warszawa, Poland; 3 Centre for Advanced Technologies, Adam Mickiewicz University in Poznań, ul. Uniwersytetu Poznańskiego 10, 61-614 Poznań, Poland; 4 Faculty of Chemistry, Adam Mickiewicz University in Poznań, ul. Uniwersytetu Poznańskiego 8, 61-614 Poznań, Poland

**Keywords:** icephobicity, hydrophobicity, ice adhesion, epoxy resin, multifunctionalized silicone compounds (MFSC), polysiloxanes

## Abstract

Anti-icing coatings have provided a very good alternative to current, uneconomic, active deicing methods, and their use would bring a number of significant benefits to many industries, such as aviation and energy. Some of the most promising icephobic surfaces are those with hydrophobic properties. However, the relationship between hydrophobicity and low ice adhesion is not yet clearly defined. In this work, chemical modification of an epoxy gelcoat with chemical modifiers from the group of double organofunctionalized polysiloxanes (generally called multifunctionalized organosilicon compounds (MFSCs)) was applied. The anti-icing properties of manufactured coatings were determined by means of measurements of shear strength between the ice layer and the modified surface, conducted using a tensile machine. In the work, tests were also performed on the roughness, wettability, and durability of the properties in an aging chamber. It was found that the performed modifications of the coating’s chemical composition by the addition of polysiloxanes enabled us to reduce ice adhesion by 51% and to increase the water contact angle by 14% in comparison to the neat gelcoat. A reduction in ice adhesion was also observed with the increasing water contact angle and with decreasing surface roughness. In addition, only one modification recorded an increase in ice adhesion after exposure in the aging chamber.

## 1. Introduction

There are several branches of industry where icing is undesirable, such as green power energy, aviation, and aerospace [1,2]. One of the methods dealing with icing on surfaces exposed to winter conditions described in the literature is the use of icephobic coatings. The icephobic properties of the surfaces of solids are associated with several essential parameters. These are as follows: the repulsion of over-saturated water droplets, a decrease in the temperature of ice formation, a long water freezing time, and low ice adhesion strength to a given surface [3]. Additionally, durability of properties is an important factor for such surfaces, since they are usually applied on structures under difficult operating conditions. They are exposed to, among others, abrasion, mechanical wear, the impact of sand or droplets, chemical contaminants, changes in ambient temperature, and prolonged exposure to sunlight [4]. The durability of icephobic surfaces is often tested by repeated icing/deicing cycles, where deicing occurs either due to ice desorption [5] or melting [6]. Degradation is reported either in terms of an increase in ice adhesion with progressive cycling [7], a decrease in wettability performance [5], or both [2]. Mobarakeh et al. proved that after cycles of icing and deicing, the icephobicity and hydrophobicity of the coatings deteriorate [8,9]. There are also works in which durable hydrophobic coatings have been produced that retain their properties even after abrasion with sandpaper [10,11].

There are various approaches to the design of icephobic coatings. The icephobic coatings developed to date can be divided into several categories that differ in matrix material and the mechanism of icing reduction [12]. These include siloxane [7], fluoropolymer [13,14], biomimetic [15], nanotube [16], metallic [17,18], surface-texturing [19], or liquid-filled coatings [15,20,21]. The most promising icephobic coatings include also a dependence on hydrophobic properties [22,23]. However, the existing hydrophobic coatings exhibit two main limitations in application: a lack of durability [24,25,26,27] and complexity of manufacturing techniques [28,29,30] and not always ensuring icephobic properties.

Currently, there is no universal and direct correlation in the literature between the strength of ice and the strength of water adhesion to the surface. The adhesion of ice and water to the surface of the solid depends on the intermolecular forces that act on the boundaries of the ice–solid and water–solid phases. It has been proven that obtaining only hydrophobic/superhydrophobic properties can be insufficient to obtain icephobic properties in some cases [31]. The water contact angle (WCA) can be the dominating mechanism for low ice adhesion on smooth surfaces but can fail on rough surfaces. Damage of surface structures and its development by deicing and interlocking between ice and the surfaces can lead to high ice adhesion. For this reason, the ice adhesion on hydrophobic surfaces should not be taken for granted and should instead be investigated thoroughly. Therefore, most of the research concerning icephobic coatings focuses, apart from hydrophobic properties, on the aspects of freezing delay or ice adhesion, which should be as low as possible.

Other approaches known from the literature to improve hydro- and icephobic properties without the significant development of surface roughness are various modifications of the chemical composition of the coating. In the present study, chemical modification of the epoxy resin-based gelcoat was performed. Some of the main chemical modifiers that are used to improve hydro- and ice repellency contain highly hydrophobic additives such as fluorined or organosilicon compounds. These coatings, with low post-surface energy, have been studied for years for minimizing ice deposition [32,33,34,35]. Kozera et al. in 2020 [36] showed that the chemical modification of a gelcoat based on unsaturated polyester resin with compounds from the MFSC group (triple functionalized polyhedral oligomeric silsesquioxanes (POSS) and double organofunctionalized polysiloxanes) enables one to significantly increase the surface wetting angle and reduce the adhesion to ice compared to the unmodified resin. Linear polysiloxanes (also called oligosiloxanes, depending on the number of structural units) have a chain structure composed of D units, where the inert group is usually the methyl group -OSiMe_2_O- (the most popular poly(dimethylsiloxane), PDMS) or, less commonly, the phenyl group, and terminated groups of type T (usually -OSiMe_3_) containing both inert and functional building units in the chain structure [37,38]. Methyl polysiloxanes and their copolymers usually occur in the form of oils, which greatly facilitates their application. Additional advantages of their wide application are the low price, high availability and wide range, good thermal stability, low surface energy, and the ability to operate in a wide temperature range [39]. At present, on the market, there are a variety of chemical components dedicated to specific classes of polymers, e.g., epoxy resins, to improve the mechanical properties or flexibility of composites [40,41,42], as a blend of epoxy and silicone resin compatibilizers [43], and as modifiers of polyester resins toward icephobic properties [36,44], as well as hydrophobic [45] and mechanical properties and impact strength [46]. The synergistic effect of the low surface energy of the functional group bonds to the chain and the low elastic modulus can result in an effective and durable improvement in the icephobic properties [12,47].

Polysiloxanes in resin systems can act as silane coupling agents, enabling the introduction of appropriate functional groups compatible with the resin matrix and capable of producing strong or weaker chemical interactions with it, as well as functional groups oriented on the material’s surface, which may be reactive (e.g., bonding with the reinforcement (reinforcing medium)) or inert, giving the desired composite properties [48,49]. In this study, the introduction of functional groups into the structure of poly (hydrosiloxane) and hydrosiloxane took place in a catalytic hydrosilylation reaction due to the presence of a reactive Si–H group [50].

In this work, an attempt was made to obtain a material with a surface exhibiting hydrophobic and anti-icing properties by the modification of an epoxy gelcoat with original in-house-synthesized chemical modifiers from the group of functionalized polysiloxanes (generally called multifunctionalized organosilicon compounds (MFSCs)) and to test the influence of various functional groups on the matrix performance. All organosilicon modifiers have been designed in such a way that they contain in their structure non-polar, hydrophobic alkyl groups and oxirane groups that are compatible with the polymer matrix used. The icephobic properties of the modified gelcoats were determined by testing the ice adhesion to the surface and the hydrophobic properties by water contact angle and contact angle hysteresis. In this work, an attempt was also made to characterize the relations between the roughness, wettability, and icephobicity. Additionally, special effort was made to evaluate the durability of the properties of the samples, after aging chamber tests.

## 2. Materials and Methods

### 2.1. Matrix Material

The starting material used to fabricate the samples was an epoxy gelcoat, ZEC, from Primson Composites. In industry, it is used as a protective coating for epoxy laminates, which are used to manufacture, among other products, wind turbine blades. The ZEC gelcoat is a high-quality white epoxy gelcoat consisting of a mixture of epoxy resin, obtained from bisphenol A and epichlorohydrin and glycidyl ethers, and auxiliary substances. It is characterized by increased scratch and UV resistance. The hardener used is a low-viscosity HP 25 V, which enables curing of the gelcoat at room temperature; 100 g of gelcoat cures at 23 °C for 30–40 min.

### 2.2. Synthesis of Chemical Modifiers

The substances used in the modifier synthesis were silicon compounds (trimethylsiloxy-terminated polymethylhydrosiloxanes (PMHS)) and olefins (hexene, octane, allyl-glycidyl ether) purchased from Linegal Chemicals Warsaw, Poland; a solvent (toluene) from Avantor Performance Materials Poland S.A. Gliwice, Poland; and chloroform-d, toluene-d8, and a Karstedt catalyst from Sigma Aldrich Poland, Poznan, Poland. In the process, toluene was dried and purified with the MB SPS 800 Solvent Drying System and stored under an argon atmosphere in Rotaflo Schlenk flasks. 

Mixtures of allyl-glycidyl ether (AGE) (0.168 mol) and hexene (HEX) (0.336 mol) in a molar ratio of 1:2, and allyl-glycidyl ether (0.168 mol) and octene (OCT) (0.336 mol) in a molar ratio of 1:2, were added to a solution of trimethylsiloxy-terminated polymethylhydrosiloxanes 992 or 991 (30 g, 0.504 mol) in toluene and allyl-glycidyl ether (0.100 mol) and hexene (0.403 mol) in a molar ratio of 1:4. The mixture was constantly stirred and heated to 70 °C. Then, Karstedt’s catalyst solution (10^−a^ eq Pt/mol SiH) was added. The reaction mixture was heated in reflux and stirred until the full conversion of Si–H (controlled by FT-IR). After confirming the complete conversion of the mixture, post-reaction evaporation was performed on a slow-speed vacuum evaporator.

### 2.3. Analysis of Chemical Modifiers

The nuclear magnetic resonance (NMR) ^1^H, ^13^C, and ^29^Si spectra were recorded at 25 °C using CDCl_3_ as a solvent by means of the Bruker Ascend 400 and Ultra Shield 300 spectrometers (both from Bruker Poland). Chemical shifts were reported in pm for 1H and 13C with reference to the residual solvent (CHCl_3_) peaks.

Fourier transform infrared (FT-IR) spectra were obtained as well. The machine used was a Nicolet iS 50 Fourier transform spectrophotometer (Thermo Fisher Scientific, Walsham, MA, USA) equipped with a diamond attenuated total reflectance (ATR) unit with a resolution of 0.09 cm^−1^. The reaction progress was controlled by FTIR spectroscopy (disappearance of the Si–H absorption band at a wavelength of 2200–2100 cm^−1^).

### 2.4. Preparation of Samples

The fabrication method of samples containing MFSC was presented in a previous paper [36]. The only difference is the type of polymer gelcoat matrix. The hardener was added to the epoxy gelcoat at a ratio of 100:25. The chemical modifiers were added in amounts of 2 wt.%. The composition of each prepared chemical modifier (core type, functional groups, and their ratios to each other) and the fabricated gelcoat samples is shown in Table 1.

### 2.5. Determination of Roughness

The roughness tests were performed using a non-contact 3D surface profilometer, the Slynx Sensofar from Sensofar Metrology (Barcelona, Spain). Two parameters were determined: the Sa parameter, i.e., the arithmetic mean height of the surface, and the Sz parameter, i.e., the height of the highest point on the surface. The final values are the average of three different measuring points on the surfaces.

In order to extend the roughness analysis, after exposing the samples in the aging chamber, the roughness of the samples was examined using a Bruker Dimension ICON XR for atomic force microscopy (AFM). Two parameters were determined: the Ra parameter, i.e., arithmetic mean profile ordinate, and the RSm parameter, i.e., mean profile element width. The final values are the average of three different measuring points on the surfaces.

### 2.6. Hydrophobicity Measurements

In the presented studies, the wettability of the surfaces was determined by measuring the water contact angle (WCA) and the contact angle hysteresis (CAH). Measurements were made using an OCA15 goniometer (DataPhysics Instruments, (Filderstadt, Germany) with OCA software. The volume of the droplets used was equal to 5 μL. The computed WCA values are the averages of three different measurement points on the surfaces.

### 2.7. Ice Adhesion Measurements

Ice adhesion (IA) measurement methods are not standardized, either in industry or in the lab tests described in the literature. The IA measurement method used in this paper was to measure the shear strength between the ice layer and the modified surface using the universal testing machine, Zwick/Roel Z050. A detailed description of the measurement method was presented in a previous paper [34]. The final IA values are the averages of five measurements.

### 2.8. Climate Aging Test

The aging tests were conducted in a climate chamber adapted to rapid temperature changes. The samples were exposed to 100 icing/deicing cycles over a temperature range from −10 °C to 25 °C. After aging, the samples were retested for their hydrophobicity, roughness, and icephobicity.

### 2.9. Gloss and Color Measurements

Colorimetric measurements were performed using an EnviSense NR60CP colorimeter for the $L^{*}a^{*}b^{*}$ parameters (also referred to as CIELAB). In the $L^{*}a^{*}b^{*}-L^{*}$ system, it represents the brightness, while $a^{*}$, $b^{*}$ are the chromaticity coordinates. The L component describes the brightness (luminance), a: the color from green to magenta, b: the color from blue to yellow.

The color difference can be calculated by using the formula

(1) $$\Delta E=\sqrt{\Delta L^{2}+\Delta a^{2}+\Delta b^{2}}$$

where $\Delta L=L_{1}-L_{2}$; $\Delta a=a_{1}-a_{2}$; $\Delta b=b_{1}-b_{2}$.

The gloss was measured based on the PN/EN ISO 2813/2014 standard. Depending on the degree of gloss of the sample surface, the following measurement angles were established:

- 20° geometry for high-gloss surfaces;
- 60° geometry for semi-gloss surfaces;
- 85° geometry for matt surfaces.

The measurements were made with a 3Color GM30 Gloss Meter.

## 3. Results

### 3.1. Characterization of Polysiloxane Derivatives

Modifiers were prepared according to the synthesis procedure described in Section 2.2. NMR and FT-IR were performed to prove the full conversion of substrates by observation of the disappearance of the characteristic signal at 2141 and 889 cm^−1^, due to stretching and bending of the Si–H group, respectively. Based on ^1^H NMR analysis, the conversion for all compounds was >99%. The structure and purity of the modifiers were also confirmed by ^1^H NMR, ^13^C NMR, and ^29^Si NMR analysis.

**Scheme 1:**
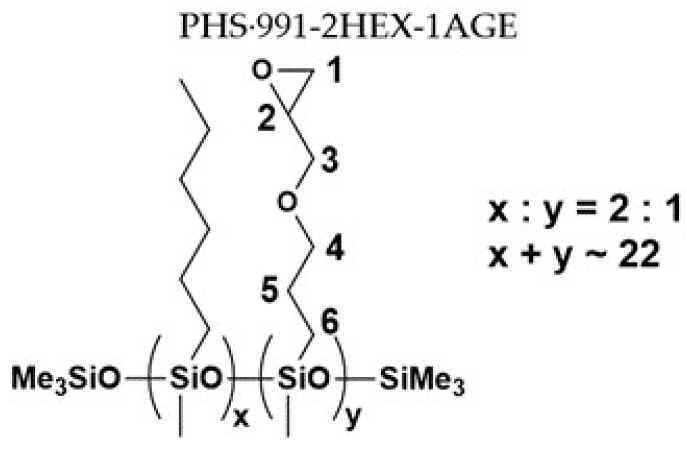
The scheme of the structure of the MFSC 1 modifier.

**Scheme 2:**
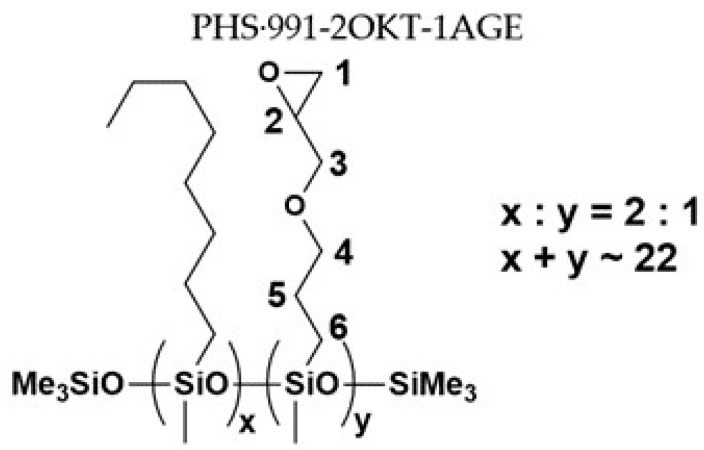
The scheme of the structure of the MFSC 2 modifier.

**Scheme 3:**
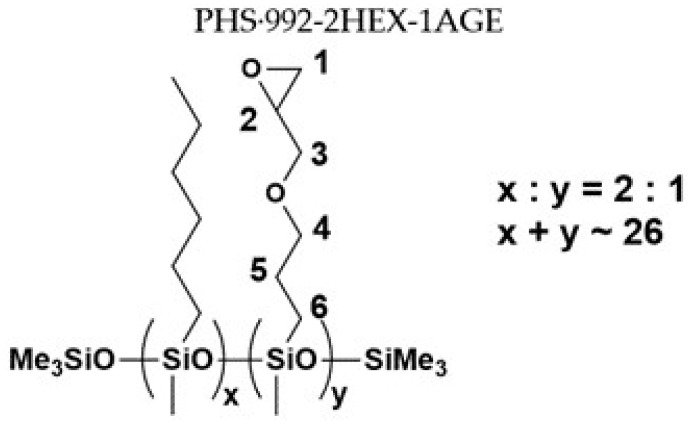
The scheme of the structure of the MFSC 3 modifier.

**Scheme 4:**
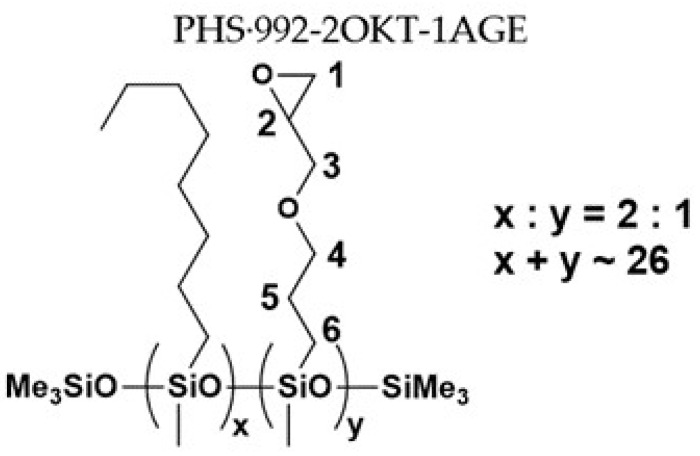
The scheme of the structure of the MFSC 4 modifier.

**Scheme 5:**
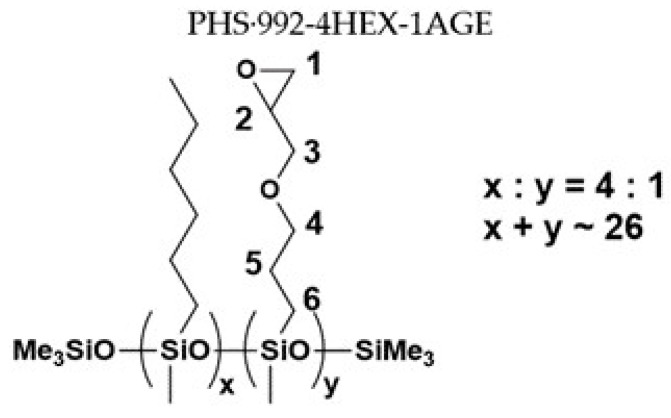
The scheme of the structure of the MFSC 5 modifier.

The product structures were confirmed by NMR spectroscopy (Scheme 1, Scheme 2, Scheme 3, Scheme 4 and Scheme 5); the following signals were assigned:

^1^H NMR (400 MHz, CDCl_3_):δ (ppm) = 3.71–3.68 (m, position 3), 3.47–3.41 (m, position 3 and 4), 3.14 (m, position 2), 2.80–2.78 (m, position 1), 2.61 (m, position 1), 1.64–1.61 (m, position 5), 1.32–1.28 (m, hexyl -CH_2_-), 0.90–0.88 (m, hexyl -CH_3_), 0.54–0.53 (m, SiCH_2_-), 0.13–0.06 (s, SiMe, SiMe_3_).

^13^C NMR (101 MHz, CDCl_3_):δ (ppm) = 74.23, 72.15, 71.53, 69.69, 50.95, 44.42 (AGE), 33.17, 31.77 (HEX), 23.35, 23.01 (AGE), 22.76, 17.70, 17.61, 17.46, 14.25 (HEX), 13.63, 13.51 (AGE), 1.98–1.51, −0.22–(−0.58) (SiMe, SiMe_3_).

^29^Si NMR (79,5 MHz, CDCl_3_):δ (ppm) = −20.98–(−22.88) (SiMe, SiMe_3_).

^1^H NMR (400 MHz, CDCl_3_):δ (ppm) = 3.72–3.68 (m, position 3), 3.50–3.40 (m, position 3 and 4), 3.17–3.13 (m, position 2), 2.82–2.79 (m, position 1), 2.63–2.60 (m, position 1), 1.69–1.62 (m, position 5), 1.34–1.30 (m, octyl -CH_2_-), 0.93–0.89 (m, octyl -CH_3_), 0.57–0.51 (m, SiCH_2_-), 0.12–0.08 (SiMe_2_).

^13^C NMR (101 MHz, CDCl_3_):δ (ppm) = 74.30, 71.53, 50.94, 44.39 (AGE), 33.63, 33.55, 32.11, 29.57, 29.50 (OCT), 23.37, 23.21 (AGE), 22.83, 17.65, 17.54, 14.22 (OCT), 13.54, 13.44 (AGE), 1.98, 1.60, −0.19, −0.29, −0.47 (SiMe, SiMe_3_).

^29^Si NMR (79,5 MHz, CDCl_3_):δ (ppm) = −21.28–(−23.45) (SiMe, SiMe_3_).

^1^H NMR (400 MHz, CDCl_3_):δ (ppm) = 3.68–3.65 (m, position 3), 3.47–3.34 (m, position 3 and 4), 3.13–3.11 (m, position 2), 2.78–2.76 (m, position 1), 2.59–2.58 (m, position 1), 1.64–1.60 (m, position 5), 1.33–1.26 (m, hexyl -CH_2_-), 0.88–0.86 (m, hexyl -CH_3_), 0.51–0.48 (m, SiCH_2_-), 0.13–0.03 (s, SiMe, SiMe_3_).

^13^C NMR (101 MHz, CDCl_3_):δ (ppm) = 74.31, 71.53, 50.97, 44.44 (AGE), 33.65, 33.57, 32.12, 29.58, 29.51, (HEX) 23.37, 23.23, 23.18, 23.10 (AGE), 22.85, 17.82, 17.66, 17.55, 14.25 (HEX).

13.66, 13.44 (AGE), 2.01, 1.61, −0.19, −0.29, −0.46 (SiMe, SiMe_3_).

^29^Si NMR (79,5 MHz, CDCl_3_):δ (ppm) = −20.99–(−21.26) (SiMe, SiMe_3_).

^1^H NMR (400 MHz, CDCl_3_):δ (ppm) = 3.70–3.67 (m, position 3), 3.47–3.41 (m, position 3 and 4), 3.14 (m, position 2), 2.79–2.78 (m, position 1), 2.61–2.60 (m, position 1), 1.66–1.60 (m, position 5), 1.34–1.27 (m, octyl -CH_2_-), 0.90–0.88 (m, octyl -CH_3_), 0.53–0.50 (m, SiCH_2_-), 0.15–0.05 (SiMe, SiMe_3_).

^13^C NMR (101 MHz, CDCl_3_):δ (ppm) = 74.35, 72.16, 71.53, 69.71, 50.96, 44.43 (AGE), 33.65, 33.58, 32.13, 29.58, 29.51 (OCT), 23.38, 23.23 23.11, (AGE), 22.85, 17.83, 17.55, 14.24 (OCT), 13.66 (AGE), 2.00, 1.61, −0.18, −0.29, −0.54 (SiMe, SiMe_3_).

^29^Si NMR (79,5 MHz, CDCl_3_):δ (ppm) = −21.24–(−22.95) (SiMe, SiMe_3_).

^1^H NMR (400 MHz, CDCl_3_):δ (ppm) = 3.70–3.67 (m, position 3), 3.51–3.40 (m, position 3 and 4), 3.15–3.13 (m, position 2), 2.80–2.78 (m, position 1), 2.62–2.60 (m, position 1), 1.68–1.62 (m, position 5), 1.34–1.30 (m, hexyl -CH_2_-), 0.92–0.89 (m, hexyl -CH_3_), 0.54–0.51 (m, SiCH_2_-), 0.11–0.07 (s, SiMe, SiMe_3_).

^13^C NMR (101 MHz, CDCl_3_):δ (ppm) = 74.35 (position 4), 71.52 (position 3), 50.95 (position 2), 44.41 (position 1), 33.29, 33.22, 31.81, 23.18, 23.06, 22.79, 17.84, 17.77, 17.57, 14.25 (-CH_2_-), 1.97, 1.61, −0.20, −0.31, −0.47 (SiMe, SiMe_3_).

^29^Si NMR (79,5 MHz, CDCl_3_):δ (ppm) = −21.28–(−21.36) (SiMe, SiMe_3_).

### 3.2. Roughness of Surface

**Figure 1:**
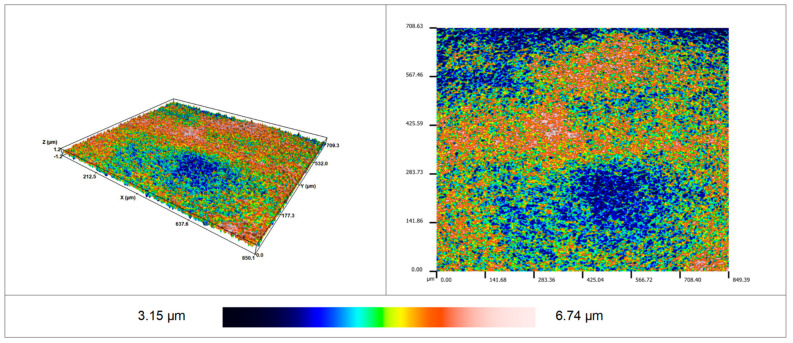
The visualization of the surface of the reference sample made with a profilometer.

In the design of icephobic surfaces, one of the most important parameters is the surface roughness. The parameters determining the surface roughness of the gelcoat samples are presented in Table 2. The reference sample (non-chemically modified epoxy gelcoat) obtained Sa equal to 55.43 nm and Sz equal to 1048 nm. Figure 1 shows a visualization of the surface of the reference sample made with the profilometer. For the modified samples, the Sa parameter values varied from 50.52 nm to 82.77 nm, while the range of Sz parameter values was from 597 nm to 1213 nm. It should be noted that all samples were fabricated to obtain the smoothest possible surfaces (samples were cast on foil-covered glass). This technique was used to analyze, as accurately as possible, the effect of chemical modification on wettability and ice adhesion and to eliminate the effect of roughness on these properties. Despite this, some of the modified samples achieved a roughness value that was different from the reference sample. This could be due to possible reactions or insufficient compatibility between the matrix and the modifiers.

A visible increase in the value of the Sa parameter was recorded compared to the reference sample for the two samples that were modified with MFSC 1 and MFSC 2 polysiloxanes. The increase in the value of this parameter occurred by 49% and 42%, respectively. For the rest of the samples, the Sa values stayed within the ranges of measurement deviation.

In the case of the Sz parameter, only samples MFSC 3 and MFSC 5 showed a noticeable change. The lowest value was achieved by the sample modified with MFSC 3; the value decreased by as much as 43% in comparison with the unmodified sample. For the remaining samples, there was no significant change and values stayed in the range of standard deviation.

### 3.3. Wetting Properties

Another important surface property that can affect the icephobicity of a surface is its hydrophobicity. It has been proven in many works that surfaces that are poorly wettable by water are able to minimize the contact time between the cooled droplet and the substrate. The surfaces that exhibit a water contact angle (WCA) higher than 90° are considered hydrophobic. Regarding the value of contact angle hysteresis (CAH), there is no clear limit to determine the hydrophobicity, but the lower its value, the more hydrophobic the surface is.

The reference sample, an epoxy gelcoat, obtained a WCA of 88°. For the tested samples that were chemically modified, the WCA value increased compared to the reference sample, i.e., the surface hydrophobicity was improved. The range of WCA values was from 91° to 100°. The highest increase in WCA value was recorded for the sample modified with MFSC 1 and MFSC 4. Compared to the reference sample, the increase occurred by 14%.

The unmodified epoxy gelcoat obtained a contact angle hysteresis (CAH) value of 31°. The range of values of this parameter for the modified samples is from 28° to 38°. Only one modified sample (MFSC 4) showed a decrease in CAH in comparison with the reference sample; the reduction was 10%. It is worth mentioning that these samples also obtained the highest WCA values among the tested samples. The sample modified with MFSC 1 possessed the same values of CAH as the reference sample. The highest CAH value was obtained by samples modified with MFSC 5; the increase was equal to 23% compared to the reference sample.

### 3.4. Ice Adhesion Strength

**Figure 2:**
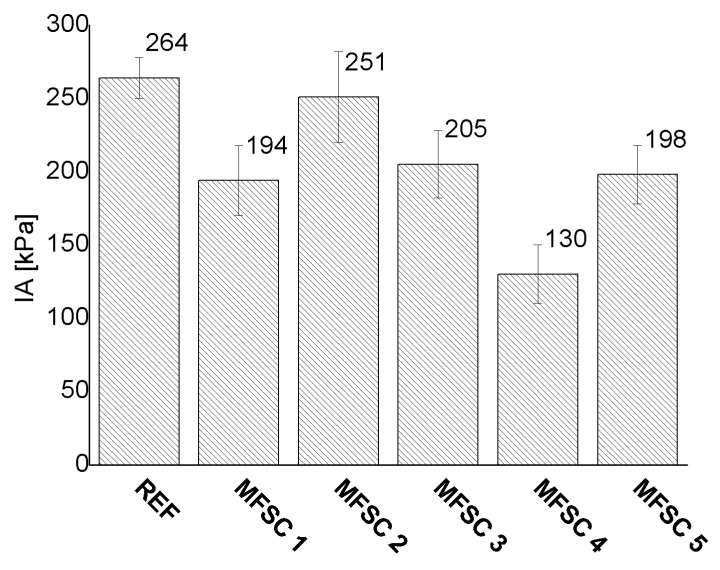
The ice adhesion of gelcoat samples.

Ice adhesion is one of the main parameters that describes the icephobicity of a surface. The lower its value, the less force is needed for the ice to break away from the surface, i.e., the surface is more icephobic. Epoxy coatings are among the surfaces where ice can easily accumulate [51], and are commonly used as coatings for many structures exposed to winter conditions, such as wind turbines. Thus, an important aspect is to improve their icephobicity. Figure 2 presents the ice adhesion values.

The reference sample, i.e., an unmodified epoxy gelcoat, achieved the IA value of 264 kPa. All chemical modifications yielded an improvement, namely causing a decrease in IA value. The range of IA values of the modified samples varied from 130 kPa to 251 kPa. The sample modified with polysiloxane MFSC 4 showed the highest decrease in ice adhesion compared to the unmodified sample. The samples modified with MFSC 1, MFSC 3, and MFSC 5 obtained similar values of IA. The MFSC 4 modifier reduced the IA by as much as 51% compared to the reference sample. It is noteworthy that the sample that obtained the best icephobic properties (the lowest IA) recorded one of the highest WCA values among the tested samples. The lowest decrease in ice adhesion was recorded by the sample modified with the MFSC 2 polysiloxane; the reduction was only 5% and stayed close to the standard deviation value.

### 3.5. Durability of Surfaces

The samples that achieved the lowest ice adhesion values after chemical modification were selected for aging tests, namely MFCS 1, 4, 5. The samples were placed in an aging chamber and subjected to 100 cycles of icing and deicing to determine their durability. After exposure in the chamber, parameters describing the icephobicity, hydrophobicity, roughness, gloss, and color of the surface were again measured.

#### 3.5.1. Ice Adhesion Strength

**Figure 3:**
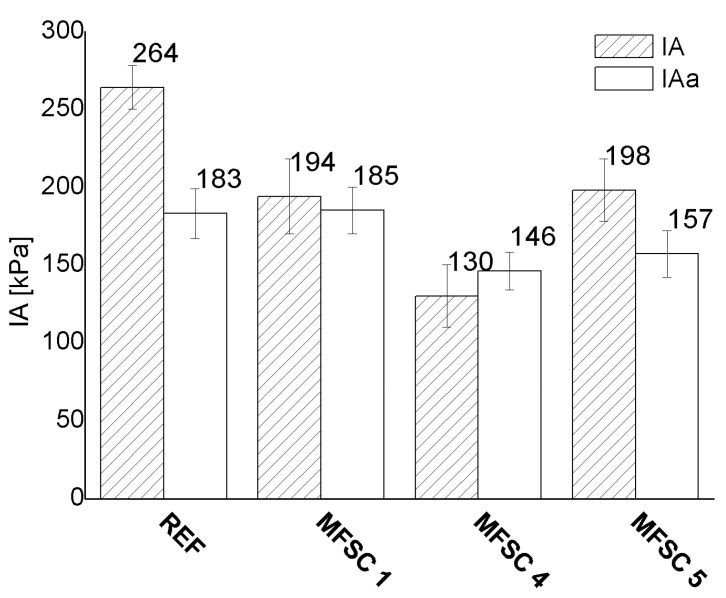
The comparison of ice adhesion before and after exposure in an aging chamber.

Figure 3 presents a comparison of values before (IA) and after the aging chamber (IAa). A significant decrease in the IAa value after the aging chamber was observed for the reference sample modified with MFSC 5. The reference sample had the highest decrease; the IAa reduction was equal to 31%. In contrast, the samples modified with MFSC 1 and MFSC 4 achieved a minimal decrease in IAa values. Considering the value of the standard deviation, it can be concluded that both samples before and after the chamber obtained the same IA value. Thus, it can be concluded that the sample modified with spherosilicate MFSC 1 was the most stable and resistant compared to the other modifiers used in this work. Only the sample modified with MFSC4 showed an increase in IAa values. However, this increase was not significant and was within the range of measurement error. A possible reason for the decrease in the ice adhesion of the reference sample after the aging chamber is presented in the Discussion section.

#### 3.5.2. Wettability

**Figure 4:**
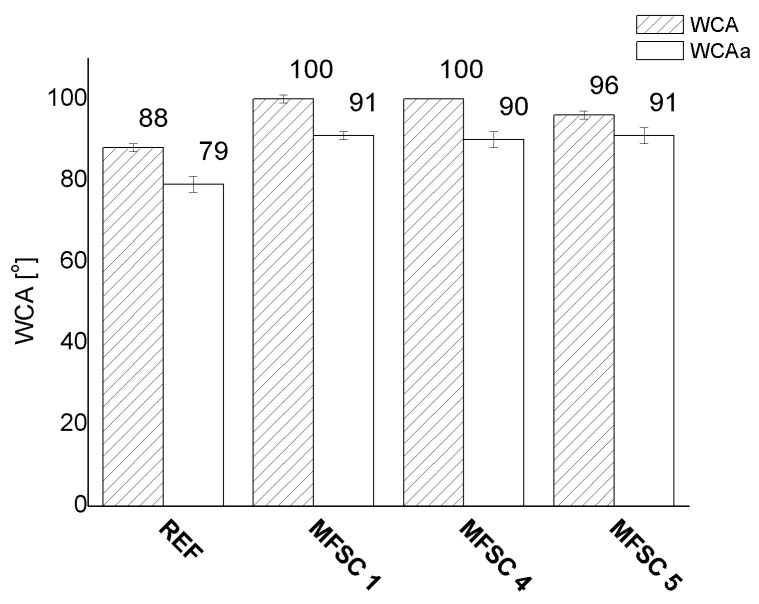
The comparison of water contact angle before and after exposure in an aging chamber.

Figure 4 presents a comparison of WCA values before and after (WCAa) the aging chamber. All samples, both modified and reference, recorded a decrease in WCAa values after exposure in the aging chamber. The lowest WCAa value was obtained for the reference sample; the WCAa was equal to 79°. The rest of the modified samples exhibited WCAa values from 90° to 91°.

The sample modified with polysiloxane MFSC 5 obtained the lowest decrease in the WCA value among the samples modified with polysiloxanes; the difference between the WCA values was 5%. This sample exhibited one of the lowest IAa results after aging and was one of two samples with the lowest IAa value after aging compared to the reference sample.

#### 3.5.3. Roughness

In order to characterize the surface topography and observe more accurate changes in the arithmetic mean profile ordinate (Ra) and mean profile element width before (RSm) and after aging (RSma), a study using atomic force microscopy (AFM) was carried out. The determined roughness parameters for the tested samples are shown in Table 3.

The reference sample after 100 cycles of icing and deicing treatment indicated lower surface roughness compared to the roughness before aging, where the Raa value decreased by 25%. A similar tendency was recorded for the sample modified with the MFSC 5 additive. The sample modified with the MFSC 1 polysiloxane remained unchanged before and after the aging chamber. In contrast, the sample modified with MFSC 4 recorded a slight increase in roughness after exposure to the chamber. Similar changes as for the Ra were observed for the values of the RSm parameter, where the reference sample presented a decrease of 26% compared to the state before aging.

**Figure 5:**
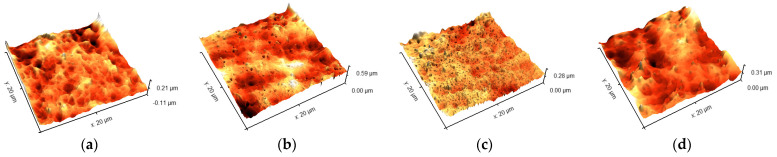
The visualization of the surfaces of the samples before aging for (**a**) unmodified sample and modified samples with (**b**) MFSC 1; (**c**) MFSC 4; (**d**) MFSC 5.

**Figure 6:**
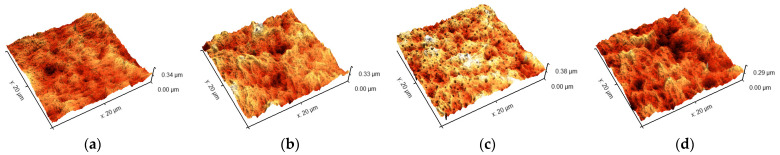
The visualization of the surfaces after aging for (**a**) unmodified sample; modified samples with (**b**) MFSC 1; (**c**) MFSC 4; (**d**) MFSC 5.

To better illustrate the changes in roughness, Figure 5 and Figure 6 show the visualization of the surface of the samples before and after 100 icing/deicing cycles. Comparing images of the surface of the reference sample, it can be seen that the surface has been smoothed, and elevations and depressions have decreased. For the sample modified with MFSC 4, for which the roughness parameters increased, the appearance after aging of larger and more pronounced depressions and elevations can be observed.

#### 3.5.4. Gloss and Color

In the case of polymeric materials, one of the first visible effects of degradation is a change in the color and the gloss of the surface; thus, these properties were tested before and after exposure in an aging chamber.

The gloss results expressed in gloss units are shown in Table 4. The surface gloss was measured at a 60° incidence angle. The addition of organosilicon modifiers resulted in a decrease in surface gloss in comparison with the unmodified sample before exposure in the aging chamber. The samples that showed the highest gloss reduction were modified with polysiloxanes MFSC 1 and MFSC 5, while the samples modified with polysiloxane MFSC 4 showed the lowest gloss reduction (not more than 3%) in comparison with the reference sample.

One hundred cycles of icing and deicing resulted in a general decrease in the surface gloss values. The highest reduction of around 9% between the gloss values before and after exposure in the chamber was observed for samples modified with polysiloxanes MFSC 4 and MFSC 5. The decrease in the gloss value for the reference sample was equal to 4%. It should be added, however, that despite differences in gloss values of several percent, these are not large changes that would indicate significant surface degradation or be visible to the naked eye.

The changes in the values of parameters L, a, b, and ΔE after chemical modifications and after the aging chamber are given in Table 5. Parameter ΔE determines the total color changes. Table 6 presents a scale of the ΔE value. Δa, Δb, and ΔL were calculated based on the difference between the modified materials and the reference.

The sample modified with MFSC 4 before aging recorded Δa < 0 with respect to the reference, which means that this sample became greener (less red). Two modified samples obtained Δb < 0, which means that the proportion of blue color increased on the surface (the proportion of yellow color decreased) compared to the unmodified sample. Parameter ΔL determines the lightness of the samples. All modified samples showed negative values of this parameter (darkened in comparison to the reference sample), but two samples, i.e., MFSC 1 and MFSC 4 modified with polysiloxanes, darkened imperceptibly because ΔL did not exceed the value of −0.1. The total color change is defined by parameter ΔE. The highest value of this parameter was recorded for the sample modified with polysiloxane MFSC 5. This sample achieved an ΔE value equal to around 2, which means a small color change (perceptible through close observation). In conclusion, it can be said that chemical modification does not result in a visible color change.

After exposure in the aging chamber, in comparison to the reference sample, samples modified with polysiloxanes MFSC 1 and MFSC 4 obtained the lowest values of ΔE, not exceeding 0.7 and indicating invisible color deviation. The sample modified with the MFSC 5 polysiloxane obtained a higher ΔE value than the above samples, but not significantly higher so as to induce a color change.

Table 7 shows the results of the color change after exposure in the aging chamber with respect to the color before aging.

The reference sample obtained a value of ΔE = 1.67 (very small color change compared to the reference sample before aging). The samples modified with MFSC 1 showed a reduction in this parameter. The other two samples obtained a higher value for the ΔE parameter compared to the reference sample, but the sample modified with MFSC 5 showed very little increase. The sample modified with the MFSC 4 polysiloxane achieved an ΔE value indicating a noticeable color change (ΔE > 2). Thus, it can be concluded that the MFSC 1 and MFSC 5 additives improve or do not deteriorate the degradation resistance of the epoxy gelcoat, and the MFSC 1 additive is the most stable under degradation during variable temperatures among the organosilicon compounds used in this study.

Summarizing the above, after 100 cycles of icing and deicing, the reference sample showed virtually undetectable color deviation. The samples modified with MFSC 1 and MFSC 5 indicated a very small color change, while the sample modified with MFSC 4 recorded a moderate color change.

## 4. Discussion

### 4.1. Roughness

The highest increase in roughness (Sa and Sz parameter values) compared to the reference sample was observed for samples modified with polysiloxanes with a PHS991 core (MFSC 1 and MFSC 2). These modifiers were dual-functionalized with AGE, HEX, and AGE, OCT functional groups, respectively. These groups have the same 1:2 molar ratio. The roughness parameters of the above samples are similar to each other, being within their error ranges. The modification with polysiloxanes that were functionalized with the same functional groups at the same molar ratio, but with a PHS992 core, resulted in significantly lower roughness parameter results. The Sa parameters differed by 39% and 23%, and the Sz parameter by 49% and 20%, respectively. The significant difference is mainly seen with modifiers with the HEX functional group, which resulted in the highest roughness, being significantly higher than that of the reference sample. In conclusion, the polysiloxanes with PHS992 cores showed significantly lower surface roughness than their counterparts with PHS991 cores.

Another important aspect is that as the number of HEX functional groups at the PHS992 core increases, the roughness of the samples increases. The sample with MFSC 5 with a molar ratio of AGE:HEX functional groups of 1:4 recorded an increase in the Sa parameter by 24%, compared to the sample with MFSC 3 with a molar ratio of AGE:HEX functional groups of 1:2. The Sz parameter also increased by as much as 41%. In summary, the roughness of samples modified with polysiloxanes with a PHS992 core was higher for the higher content of HEX functional groups.

### 4.2. Wettability

It cannot be clearly determined which core (PHS991 or PHS992) gives a better hydrophobicity improvement. However, it can be observed that functionalization with AGE:HEX groups gives better results for samples modified with polysiloxanes with a PHS991 core, and functionalization with AGE:OCT groups gives better results for samples modified with polysiloxanes with a PHS992 core. In both cases, the obtained increase in WCA values by modifiers having HEX and OCT functional groups compared to the reference sample is 10%. Comparing the modifiers MFSC 3 and MFSC 5, which differ in the molar ratio of AGE:HEX functional groups, it can be concluded that the modifier with a ratio of 1:4 (MFSC 5) causes a higher increase in the WCA value than the modifier with a ratio of 1:2 (MFSC 3). The wettability of a surface depends on its functionalization as well as its chemical composition. An important factor determining wettability is the presence of groups with different polarities [52]. Alkyl chains (e.g., hexyl or octyl groups) are non-polar, which increases the WCA value. Both the chain length and the percentage of non-polar groups in relation to the total weight of the compound can affect the wettability properties.

### 4.3. Ice Adhesion Strength

A higher decrease in the IA parameter among samples modified with polysiloxanes with a PHS991 core was achieved by the sample modified with polysiloxane functionalized with AGE and HEX functional groups (MFSC 1); the decrease was 27% in comparison to the reference sample. Functionalization of polysiloxanes with AGE and OCT groups resulted in only a 5% decrease in IA (MFSC 2). An inverse correlation could be observed for samples modified with polysiloxanes with a PHS992 core. A higher improvement in the icephobicity parameter was observed for the sample that was modified with AGE and OCT functional groups. The functionalization of polysiloxanes with a PHS992 core with AGE and HEX groups resulted in a higher reduction in the IA value using a molar ratio of 1:4. However, the IA value was not significantly different from that of the samples modified with a molar ratio of 1:2 functional groups, and was within the range of measurement deviation. By comparing the effect of the polysiloxane core type on the improvement in icephobic properties, it can be generally concluded that the PHS992 core is the more effective one. The sample modified with the PHS992 core polysiloxane with AGE and OCT functional groups (MFSC 4) obtained significantly lower ice adhesion than the sample modified with the PHS991 core polysiloxane with the same functional groups (MFSC 2). On the other hand, the PHS992 core polysiloxane functionalized with AGE and HEX groups (MFSC 5) resulted in a similar IA value as the PHS991 core polysiloxane (MFSC 3).

### 4.4. Ice Adhesion/Wettability/Roughness

**Figure 7:**
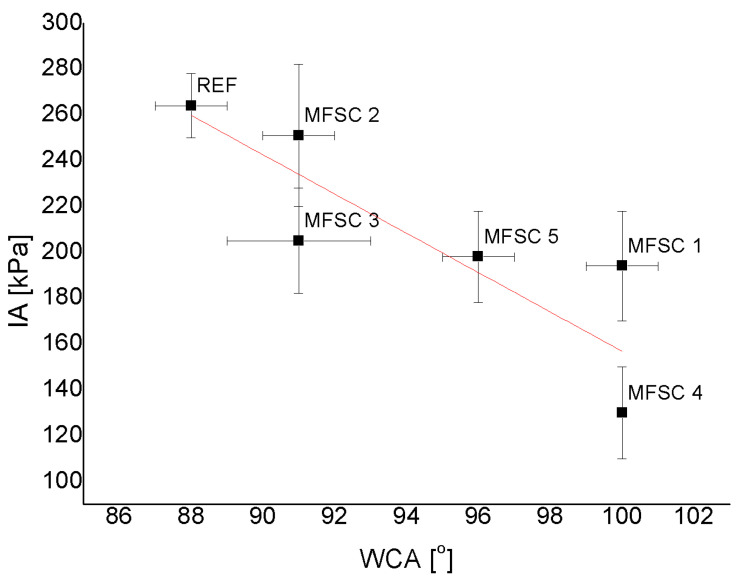
The relationship between ice adhesion and water contact angle.

**Figure 8:**
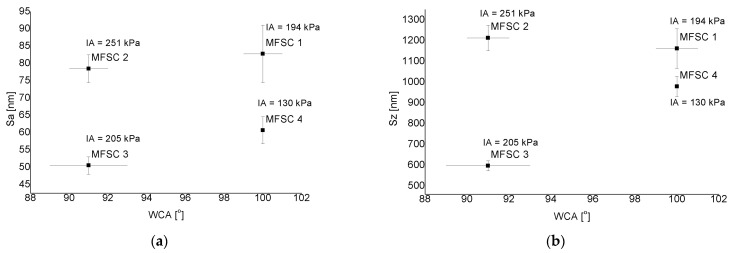
The relationship between water contact angle and (**a**) Sa, (**b**) Sz.

Figure 7 shows the correlation between the water contact angle and ice adhesion. It can be concluded that as the value of WCA increases, i.e., as the hydrophobicity of the surface increases, the value of ice adhesion decreases, i.e., the icephobic properties improve. However, it should be added that the WCA value does not exceed 100° (the surface has a slightly hydrophobic surface). The reference sample, which is the only one to achieve WCA < 90°, has the highest IA value. Interpreting the graph, it can also be seen that some samples, despite achieving the same WCA value, obtained different IA values. This is probably due to the influence of chemical groups present in the matrix. Figure 8 shows the relationship between the water contact angle and roughness parameters for samples that recorded the same water contact angle values but different ice adhesion. It can be seen that samples that obtained lower values of the Sa and Sz parameters recorded lower values of ice adhesion. In many works, the following relationship has been observed: higher surface roughness leads to greater ice adhesion. The high roughness can affect the adhesion strength of the ice by causing a mechanical interlock between the ice and the substrate [12].

### 4.5. Durability

#### 4.5.1. Ice Adhesion Strength

The two samples that achieved lower ice adhesion values after 100 icing/deicing cycles compared to the reference sample were modified polysiloxanes with a PHS992 core. The sample modified with polysiloxanes with a PHS991 core achieved a minimal increase in ice adhesion values compared to the reference sample, within the range of measurement error. The surfaces of these samples retained low surface energy after aging, resulting in low ice adhesion values. The exposure in the aging chamber may have caused some changes in the surface chemistry and consequently changed some properties that affected the icephobicity of the surface.

#### 4.5.2. Wettability

All tested samples after exposure in the aging chamber showed a decrease in the WCAa values compared to the WCA values before exposure in the aging chamber. However, when comparing the modified samples with the reference sample after exposure in the aging chamber, it can be seen that the modified samples have higher WCA values than the reference sample. Zhao et al. also observed a decrease in the water contact angle value after icing/deicing cycles [35]. The study proved that after returning to room temperature, the surface does not fully retain its hydrophobic properties and the cyclic temperature variation (from 25 °C to −10 °C) effects a gradual decrease in the WCA value.

#### 4.5.3. Roughness

A significant decrease in roughness after exposure in the aging chamber in addition to the reference sample was observed for the sample modified with the polysiloxane with a PHS992 core with AGE:HEX (1:4) functional groups (MFSC 5). This sample, as with the reference sample, also showed a decrease in IA values after aging. Smoothing the surface resulted in reduced ice adhesion (the explanation is given in Section 4.4). On the other hand, the sample modified with the polysiloxane with a PHS992 core functionalized with AGE:OCT functional groups (1:2) (MFSC 4) showed the highest increase in roughness values after 100 icing/deicing cycles. The highest stability of surface roughness (slight difference in Ra and RSm values before and after aging) was observed for the sample modified with the polysiloxane with a PHS991 core with AGE:HEX functional groups in a 1:2 molar ratio. In summary, the type of core, the functional groups, and the molar ratio may drastically affect the surface topography after aging.

## 5. Conclusions

- The highest surface roughness of the epoxy gelcoat after chemical modification was obtained for samples modified with polysiloxanes with a PHS991 core functionalized with AGE, HEX and AGE, OCT functional groups, respectively, with the same molar ratios. It is likely that the additive core and the presence of the AGE group significantly increased the roughness of these samples, even though all of them were produced within the same methodology. In general, the roughness of samples with modified polysiloxanes was increased with higher content of HEX functional groups.
- The sample modified with the polysiloxane functionalized with AGE and OCT groups with a molar ratio of 1:2 recorded the best icephobic properties. Moreover, this sample was one of two that obtained the highest WCA value. The other samples achieved a significantly lower reduction in IA values compared to the reference sample. It can also be concluded that the PHS992 core improves the icephobic properties more effectively compared to the PHS991 core.
- The following correlation was observed: as the value of the water contact angle increases, the value of ice adhesion decreases. The conducted studies corroborated the well-established assumption that ice adhesion closely depends on the surface roughness, which can lead to the formation of a mechanical blockage between the ice and the substrate.
- The highest stability of surface roughness after aging was observed for the sample modified with the polysiloxane with a PHS991 core with AGE:HEX functional groups in a 1:2 molar ratio. No noticeable color or visible gloss changes were observed.
- Cycles of icing and deicing caused a decrease in the value of the water contact angle. However, the chemical surface modification enabled the retention of its hydrophobicity after aging, despite a decrease in WCA.
- Exposure in the aging chamber did not increase ice adhesion, i.e., deterioration of the icephobic properties, while, in some cases, e.g., the reference sample, which was not chemically modified, we observed a decrease in IA after a decrease in WCA. For the rest of the samples, despite the deterioration of the WCA, the IA remained without significant changes, thus indicating the positive influence of modification on the surface stability and the possible influence of a low WCA as the reason for the IA decrease in the reference sample.

## Figures and Tables

**Table 1:** Compositions of the prepared epoxy gelcoat samples and their modifiers.

Sample No.	MFSC Type	PHS	Olefin 1	Olefin 2	Molar Ratio
1 (REF)	-	-	-	-	-
2	MFSC 1/2 wt.%	PHS991	AGE	HEX	1:2
3	MFSC 2/2 wt.%	PHS991	AGE	OCT	1:2
4	MFSC 3/2 wt.%	PHS992	AGE	HEX	1:2
5	MFSC 4/2 wt.%	PHS992	AGE	OCT	1:2
6	MFSC 5/2 wt.%	PHS992	AGE	HEX	1:4

**Table 2:** The surface parameters of gelcoat samples.

Sample No.	MFSC Type	Sa (nm)	±Sa (nm)	Sz (nm)	±Sz (nm)	WCA (°)	±WCA (^o^)	CAH (°)
1 (REF)	-	55.43	5.46	1048	55	88	1	31
2	MFSC 1/2 wt.%	82.77	8.31	1162	95	100	1	31
3	MFSC 2/2 wt.%	78.49	4.00	1213	61	91	1	34
4	MFSC 3/2 wt.%	50.52	2.50	597	24	91	2	32
5	MFSC 4/2 wt.%	60.68	3.89	979	48	100	0	28
6	MFSC 5/2 wt.%	62.42	3.12	840	38	96	1	38

**Table 3:** The roughness parameters obtained by AFM before and after aging.

Sample No.	MFSC Type	Ra (nm)	±Ra (nm)	Raa (nm)	±Raa (nm)	RSm (nm)	±RSm (nm)	RSma (nm)	±RSma (nm)
1 (REF)	-	33.38	5.41	25.15	2.06	43.40	6.92	32.02	2.73
2	MFSC 1/2 wt.%	39.96	5.73	39.83	8.40	51.16	6.98	49.90	10.76
5	MFSC 4/2 wt.%	25.21	2.65	31.54	6.97	33.20	3.35	42.25	10.07
6	MFSC 5/2 wt.%	41.99	3.06	35.54	4.41	55.28	4.00	44.87	4.63

**Table 4:** Gloss values of tested samples before and after exposure in the aging chamber.

Sample No.	MFSC Type	Before	After
1 (REF)	-	90.3	86.6
2	MFSC 1/2 wt.%	81.0	78.6
5	MFSC 4/2 wt.%	88.1	79.8
6	MFSC 5/2 wt.%	83.0	75.6

**Table 5:** ΔL, Δa, Δb values and color ($\Delta E^{*}$) changes before and after aging.

Sample No.	MFSC Type	Color before Aging				Color after Aging			
		Δ*a* *	Δ*b* **	Δ*L* ***	Δ*E*	Δ*a* *	Δ*b* **	Δ*L* ***	Δ*E*
1 (REF)	-	0.00	0.00	0.00	0.00	0.00	0.00	0.00	0.00
2	MFSC 1/2 wt.%	0.74	−0.18	−0.07	0.76	−0.53	0.42	−0.04	0.68
5	MFSC 4/2 wt.%	−0.30	0.36	−0.03	0.47	0.05	0.2	−0.58	0.62
6	MFSC 5/2 wt.%	1.62	−0.35	−1.10	1.99	−0.76	0.61	−0.26	1.01

* Δa = $a_{1}$ (sample 2, 5, or 6) − a  (reference). ** Δb = *b*_1_ (sample 2, 5, or 6) − b (reference). *** ΔL = $L_{1}$ (sample 2, 5, or 6) −  L (reference).

**Table 6:** Scale of the ΔE value.

Delta E	Perception
0 < Δ*E* < 1	Not perceptible by the human eye
1 < Δ*E* < 2	Perceptible through close observation
2 < Δ*E* < 3.5	Average color deviations, recognizable by an inexperienced observer
3.5 < Δ*E* < 5	Significant color deviations
Δ*E* > 5	Noticeable at a glance

**Table 7:** ΔL, Δa, Δb values and color ($\Delta E^{*}$) changes after aging.

Sample No.	MFSC Type	Color after Aging			
		Δa *	Δb **	ΔL ***	ΔE
1 (REF)	-	0.93	−0.22	−1.37	1.67
2	MFSC 1/2 wt.%	−0.34	0.38	−1.34	1.43
5	MFSC 4/2 wt.%	1.28	−0.38	−1.92	2.34
6	MFSC 5/2 wt.%	−1.45	0.74	−0.53	1.71

* Δa = $a_{1}$ (sample after aging) − a (sample before aging). ** Δb = $b_{1}$ (sample after aging) − b (sample before aging). *** ΔL = $L_{1}$ (sample after aging) − L (sample before aging).
